# A gp41 HR2 residue modulates the susceptibility of HIV-1 envelope glycoproteins to small molecule inhibitors targeting gp120

**DOI:** 10.1128/jvi.02267-24

**Published:** 2025-03-27

**Authors:** Debashree Chatterjee, Ling Niu, Halima Medjahed, Shilei Ding, Mehdi Benlarbi, Étienne Bélanger, Jérémie Prévost, Hung-Ching Chen, William D. Tolbert, Amos B. Smith, Marzena Pazgier, Andrés Finzi

**Affiliations:** 1 Centre de Recherche du CHUM, Montreal, Québec, Canada; 2 Infectious Diseases Division, Department of Medicine, Uniformed Services University of the Health Sciences 1685, Bethesda, Maryland, USA; 3 Département de Microbiologie, Infectiologie et Immunologie, Université de Montréal 236710 , Montreal, Québec, Canada; 4 Department of Chemistry, School of Arts and Sciences, University of Pennsylvania 312071 , Philadelphia, Pennsylvania, USA; Icahn School of Medicine at Mount Sinai, New York, New York, USA

**Keywords:** HIV, Env, gp120, gp41, CRF01_AE, temsavir, CD4mc

## Abstract

One characteristic of the HIV-1 CRF01_AE strain is that it contains a bulkier histidine residue at position 375 (H375) in its envelope glycoproteins (Env). This residue is part of the Phe43 cavity, where residue 43 of CD4 engages with gp120. It has been shown that H375 contributes to resistance against small molecule inhibitors targeting gp120. Residue 375 co-evolved with a few residues of the gp120 inner domain layers, and together they modulate the susceptibility of Env to small molecule gp120 inhibitors. Since residue 629 within the HR2 region of gp41 has also been proposed to have co-evolved with residue 375, we explored its role in the susceptibility of HIV-1 Env to two classes of small molecule gp120 inhibitors: the conformational blocker temsavir and the CD4-mimetic (CD4mc) BNM-III-170. Reversion of CRF01_AE isoleucine to a major clade methionine at position 629 had a significant but opposite impact on the susceptibility of the virus to temsavir and BNM-III-170. Mechanistically, this is associated with the capacity of residue 629 to modulate Env stability, as attested by its impact on cold inactivation. Overall, our results show how a single residue of HR2 contributes to the overall Env trimer stability and its susceptibility to gp120-targeted small molecule inhibitors.

**IMPORTANCE:**

CRF01_AE envelope glycoproteins (Env) have a well-conserved histidine at position 375. This residue is key in modulating the susceptibility of HIV-1 to small molecule Env inhibitors. Here, we report that a residue of the gp41 HR2 region affects Env trimer stability and its susceptibility to gp120-directed small molecule inhibitors. This work adds to our understanding of HIV-1 Env resistance to small molecule inhibitors.

## INTRODUCTION

The HIV-1 envelope glycoproteins (Env) are composed of a trimer of three gp120 exterior and three gp41 transmembrane subunits. These subunits are linked together by non-covalent interactions, enabling Env to undergo conformational changes during the entry process (1–4). Upon binding of gp120 to the CD4 receptor, a series of conformational changes occur within Env, leading to its interaction with the CCR5/CXCR4 coreceptors (5). Engagement of both CD4 and coreceptor enables the insertion of the fusion peptide into the target cell membrane, followed by the formation of the six-helix bundle composed of the HR1 and HR2 regions of gp41, which drives the fusion of the viral and host plasma membranes (6, 7).

HIV-1 Env is in constant evolution, posing a significant challenge to the development of an effective HIV vaccine and contributing to antiretroviral drug resistance. Despite its variability, a few regions within Env remain highly constant, including the Phe43 cavity, where phenylalanine 43 of CD4 engages gp120 (8). This site is targeted by small molecule Env inhibitors, including conformational blockers, such as temsavir (9, 10) but also by molecules that prematurely trigger conformational changes, such as small molecule CD4-mimetics (CD4mcs) (11).

Most known HIV-1 strains are susceptible to the entry inhibitor temsavir, with the notable exception of CRF01_AE (12–15). This strain originated in Central Africa and fueled the epidemic in Southeast Asia, after first appearing in Thailand in 1988 (16, 17). CRF01_AE resistance to temsavir was mapped to the presence of a histidine residue at position 375 of the Phe43 cavity (15, 18, 19). It has been previously shown that residue 375 co-evolved with the gp120 inner domain layers to bind CD4 (18, 20), but it also modulates Env susceptibility to small-molecule entry inhibitors (19, 21). Interestingly, in the initial analysis describing the co-evolution of residue 375 with Env regions, it was described that beyond the gp120 inner domain layers (residues 61, 105, 108, 474, 475, and 476), there were additional amino acids (residues 341, 430, 519, and 629) that were significantly associated with residue 375 (18).

Here, we evaluated whether these residues modulated the susceptibility of Env to small molecule gp120 inhibitors with opposite activities, i.e., a conformational blocker (temsavir) (19) versus BNM-III-170, a CD4mc, which prematurely triggers Env to downstream conformations (22).

## MATERIALS AND METHODS

Materials and methods have been previously reported in references 18–24 and are summarized below.

### Sequence analysis

The Logo plots for HIV were made using the Analyze Align tool from the HIV database and are based on the WebLogo 3 program (https://www.hiv.lanl.gov/content/sequence/ANALYZEALIGN/analyze_align.html). The 2024 Los Alamos database-curated Env alignment was used, which contains 6,669 amino acid HIV-1 group M sequences (including 942 sequences of CRF01_AE, 290 of subtype A, 3,043 of subtype B, and 1,779 of subtype C). The relative height of each letter within individual stacks represents the frequency of the indicated amino acid at that position. The numbering of all the Env amino acid sequences is based on the prototypic HXBc2 strain of HIV-1, where one is the initial methionine (23).

### Cell lines

HEK293T human embryonic kidney (obtained from ATCC, Catalog# CRL-3216), Cf2Th canine thymocytes, and TZM-bl cell lines (NIH HIV Reagent Program) were maintained at 37°C under 5% CO_2_ in Dulbecco’s Modified Eagle Medium (DMEM) (Wisent), supplemented with 5% fetal bovine serum (FBS) (VWR) and 100 U/mL penicillin/streptomycin (Wisent). Cf2Th cells stably expressing human CD4 and CCR5 (Cf2Th-CD4/CCR5, obtained from Dr. Joseph Sodroski) (25) were grown in medium supplemented with 0.4 mg/mL of G418 sulfate (Thermo Fisher Scientific) and 0.2 mg/mL of hygromycin B (Roche Diagnostics). The TZM-bl cell line is a HeLa cell line stably expressing high levels of CD4 and CCR5 and possessing an integrated copy of the luciferase gene under the control of the HIV-1 long terminal repeat (26).

### Site-directed mutagenesis

Individual or combination of mutations were introduced into the different Env expressing plasmids of CRF01_AE CM244 (18). Site-directed mutagenesis was performed using the QuikChange II XL site-directed mutagenesis protocol (Stratagene), and the required mutations were confirmed by automated DNA sequencing. The numbering of the CRF01_AE CM244 envelope glycoprotein amino acid residues is based on that of the prototypic HXBc2 strain of HIV-1, where one is the initial methionine (23).

The wild-type (WT) CRF01_AE CM244 Env contains the following residues: H375, A341, A430, I519, and I629. We mutated the following residues alone or in combination in the WT backbone: A341T, A430V, I519F, and I629M, while maintaining H375. The H375S (HS) variants incorporate a substitution of histidine to serine at position 375 along with single or combined mutations at A341T, A430V, I519F, and I629M. The layer mutation (LM) consists of the following changes: H61Y, Q105H, V108I, N474D, I475M, and K476R, with additional single or combined substitutions (A341T, A430V, I519F, and I629M). The LMHS variants integrate the LM mutations, H375S, and single or combined substitutions of A341T, A430V, I519F, and I629M.

### Small molecule gp120 inhibitors

The CD4 mimetic compound (CD4mc) BNM-III-170 was developed and synthesized as described previously (22, 27). The HIV-1 attachment inhibitor temsavir (BMS-626529) was purchased from APExBIO. The compounds were dissolved in dimethyl sulfoxide (DMSO) at a stock concentration of 10 mM, aliquoted, and stored at −80°C until further use.

### Immunoprecipitation of envelope glycoproteins

As previously described (18), using a standard calcium phosphate method, 3 × 10^5^ HEK293T cells were transfected with a pcDNA3.1 vector expressing CM244 gp160 WT or mutant variants. One day after transfection, cells were metabolically labeled for 16 h with 100 μCi/mL [^35^S] methionine-cysteine (^35^S protein labeling mix; Perkin-Elmer) in Dulbecco’s modified Eagle’s medium lacking methionine and cysteine and supplemented with 5% dialyzed fetal bovine serum. Cells were subsequently lysed in radioimmunoprecipitation assay (RIPA) buffer (140 mM NaCl, 8 mM Na_2_HPO_4_, 2 mM NaH_2_PO_4_, 1% NP-40, and 0.05% sodium dodecyl sulfate [SDS]). Precipitation of radiolabeled CM244 envelope glycoproteins from cell lysates or medium was performed with a mixture of plasma from people living with HIV. The association index is a measure of the ability of the mutant gp120 to remain associated with the Env trimer complex on the expressing cell, relative to that of the wild-type Env trimer. The association index is calculated as follows: association index = ([mt gp120] _cell_ × [wt gp120] _supernatant_)/([mt gp120]_supernatant_ × [wt gp120]_cell_), where mt is mutant and wt is wild type. The processing index is a measure of the conversion of the mutant gp160 Env precursor to mature gp120 relative to that of the wild-type Env trimer. The processing index was calculated by the following formula: processing index = ([total gp120]_mt_ × [gp160] _wt_)/([gp160]_mt_ × [total gp120]_wt_), where mt is mutant and wt is wild type. All samples were loaded onto polyacrylamide gels and analyzed by autoradiography using a TYPHOON system (Amersham).

### Recombinant luciferase viruses

Recombinant viruses containing the firefly luciferase gene were produced by calcium phosphate transfection of 293T cells with the HIV-1 proviral vector pNL4.3 Env-Luc and the plasmid expressing the wild-type or mutant of CM244 envelope glycoproteins at a ratio of 2:1. Two days after transfection, the cell supernatants were harvested and centrifuged for 5 min at 3,000 rpm to pellet cell debris. The reverse transcriptase activity was measured for all viral preparations as described previously (28). The virus-containing supernatants were stored in aliquots at -80°C.

### Cell-to-cell fusion

As previously described (18), 3 × 10^5^ 293T cells were co-transfected by the calcium phosphate method with an HIV-1 Tat-expressing plasmid, pLTR-Tat, and the CM244 wild-type or mutant envelope glycoproteins in an expression vector. After 48 h of transfection, 3 × 10^4^ 293T cells were added to TZM-bl target cells that were seeded at a density of 3 × 10^4^ cells/well in 96-well luminometer-compatible tissue culture plates (PerkinElmer) 24 h before the assay. Cells were co-incubated for 6 h at 37°C, and then lysed by the addition of 30 μL of passive lysis buffer (Promega) and three freeze–thaw cycles. An LB 941 TriStar luminometer (Berthold Technologies) was used to measure the luciferase activity of each well after the addition of 100 μL of luciferin buffer (15 mM MgSO_4_, 15 mM K_3_PO_4_ [pH 7.8], 1 mM ATP, and 1 mM dithiothreitol) and 50 μL of 1 mM D-luciferin potassium salt (Prolume).

### Virus neutralization assay

Twenty-four hours before infection, Cf2Th-CD4/CCR5 target cells were seeded at a density of 1 × 10^4^ cells/well in 96-well luminometer-compatible tissue culture plates (PerkinElmer). Then, 100 µL final volume of luciferase-expressing recombinant viruses (10,000 reverse transcriptase units) with the indicated concentration of BNM-III-170 or temsavir was incubated for 1 h at 37°C and added to the target cells. After 48 h of incubation at 37°C, the medium was removed from each well, and the cells were lysed by the addition of 30 µL of passive lysis buffer (Promega) followed by three freeze–thaw cycles. An LB 942 TriStar luminometer (Berthold Technologies) was used to measure the luciferase activity of each well after the addition of 100 µL of luciferin buffer as previously described. The neutralization half-maximal inhibitory concentration (IC_50_) value represents the concentration of compound, either BNM-III-170 or temsavir, that is required to inhibit 50% of the infection of Cf2Th-CD4/CCR5 cells by recombinant luciferase-expressing HIV-1 bearing the indicated Env.

### Cold-inactivation assay

To assess the effect of cold on virus infectivity, pseudoviruses containing wild-type or mutant envelope were produced, aliquoted, and frozen at −80°C. Cf2Th-CD4/CCR5 target cells, seeded at a density of 1 × 10^4^ cells/well in 96-well culture plates (PerkinElmer) were infected 24 h later with viruses incubated on ice for different periods of time (0, 6, 24, 48, and 72 h). After 48 hrs of infection, luciferase activity from infected cells was measured as described above. Infectivity was normalized to the one obtained at time 0 h. The inactivation half-maximal inhibitory time (t1/2) represents the time on ice needed to inhibit 50% of the infection of Cf2Th-CD4/CCR5 cells by pseudoviruses as previously described (24).

### Statistical analyses

Statistics were analyzed using GraphPad Prism version 9.4.1 (GraphPad). Every data set was tested for statistical normality, and this information was used to apply the appropriate (parametric or nonparametric) statistical test. *P* values of 0.05 were considered significant.

## RESULTS

### Comparison of the Phe43 cavity and coevolving residues among HIV-1 clades

**Fig 1:**
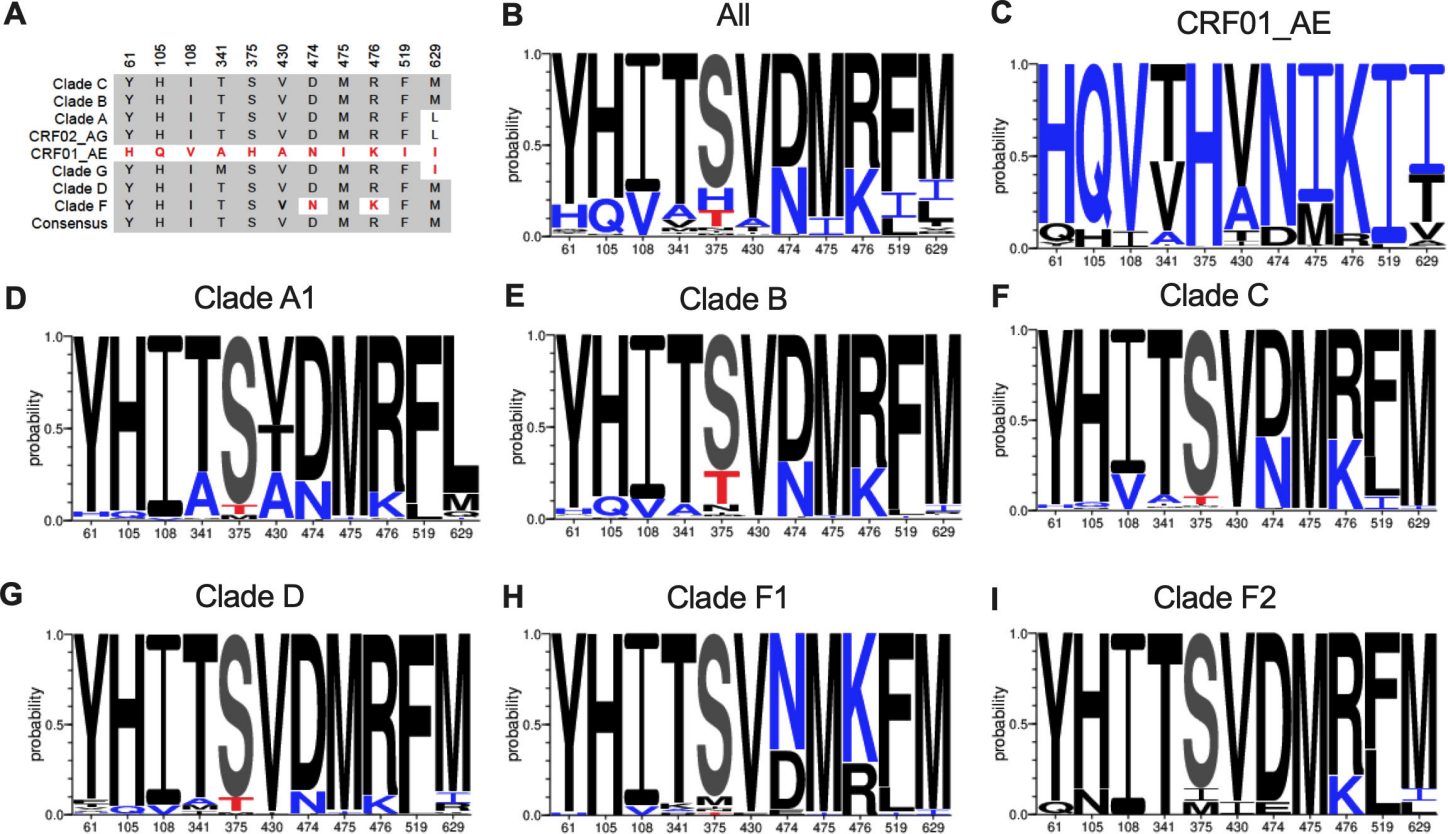
Phe43 cavity and HIV-1 *env* sequences. (A) Sequence alignment of gp120 Phe43 cavity and specific residues of gp120 and gp41 domain of different clades of HIV-1 isolates based on the Env consensus sequence of the HIV-1 group M major subtypes (clades A, B, C, D, F, G, CRF01_AE, and CRF02_AG). The 2024 Los Alamos database-curated Env alignment was used as the basis for this figure, which contains 6,669 amino acid HIV-1 group M sequences (including 942 sequences of CRF01_AE, 290 of subtype A, 3,043 of subtype B, and 1,779 of subtype C). Residue numbering is based on that of the HXBc2 strain of HIV-1. Identical residues are shaded in dark gray, and nonidentical residues are highlighted in red. (B–I) Logo depictions of the frequency of each amino acid shown at positions 61, 105, 108, 341, 375, 430, 474, 475, 476, 519, and 629 in all HIV-1 isolates (B) or in isolates from CRF01_AE (C), clade A1 (D), clade B (E), clade C (F), clade D (G), clade F1 (H), and clade F2 (I). The height of the letter indicates its frequency within the clade. S375 is shown in gray, and its coevolving residues are shown in black; H375 and its coevolving residues are shown in blue, and T375 is shown in red.

HIV-1 is classified in four major groups, M, N, O, and P. Group M viruses are responsible for most of the global HIV-1 pandemic, which is composed of nine major subtypes and circulating recombinant forms (CRFs) (21, 29–33). As reported previously (21), the gp120 Phe43 cavity has a well-conserved serine residue at position 375 in group M viruses, with the notable exception of CRF01_AE strains in which a histidine (H375) is highly conserved (Fig. 1A). We recently identified a set of six residues within the gp120 inner domain layers 1, 2, and 3 (at Env positions 61, 105, 108, 474, 475, and 476) that coevolved with residue 375 to facilitate the interaction with CD4 (collectively named LM, for layer mutants) (18, 21). These residues were also shown to be involved in Env sensitivity to CD4mc, CD4-binding site antibodies (21) and the conformational blocker temsavir (19, 21). Interestingly, in the initial work where we described residues that covary with the nature of residue 375 (18), we also reported that residues A341, A430, I519, and I629 were significantly associated with residue 375 (Fig. 1A through I). Among these, residues A341 and A430 are located in the gp120 constant 3 and 4 regions, respectively; I519 in the fusion peptide and I629 within the HR2 region of gp41. However, whether these residues can also modulate the susceptibility of Env to small-molecule entry inhibitors remains unknown.

### Residue 629 in HR2 modulates the susceptibility of Env to small molecule gp120 inhibitors

**Fig 2:**
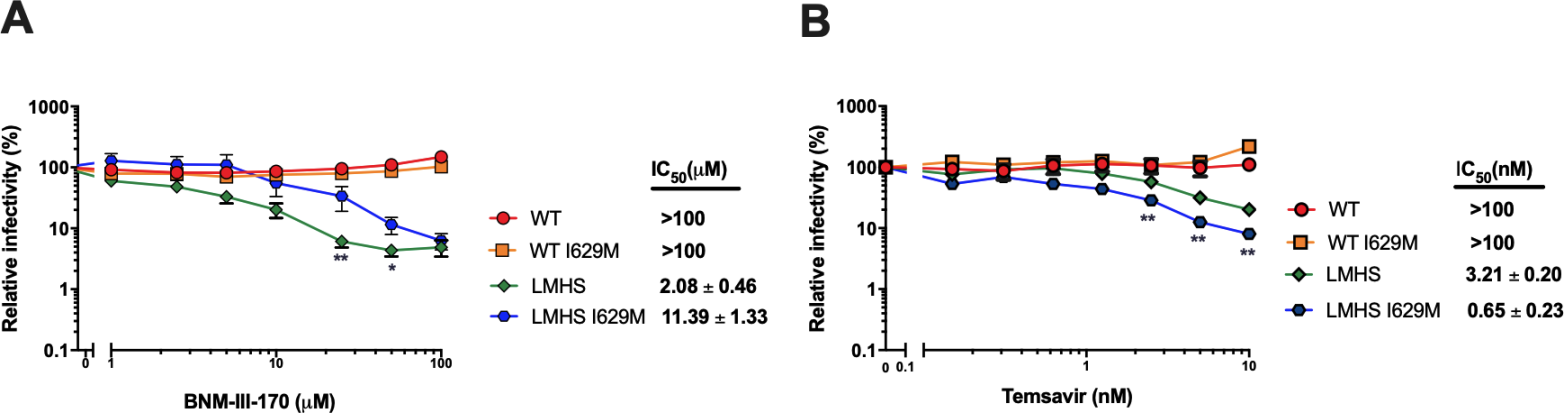
Impact of CM244 envelope variants on CD4mc and temsavir neutralization sensitivity. Recombinant HIV-1 strains expressing luciferase and bearing wild-type or mutant CRF01_AE CM244 Envs were normalized by reverse transcriptase activity. Normalized amounts of viruses were incubated with different concentrations of CD4mc BNM-III-170 (A) or temsavir (B) at 37°C for 1 h prior to infection of Cf2Th-CD4/CCR5 cells. Infectivity at each dilution of the CD4mc or temsavir is shown as the percentage of infection without the compound for each mutant. Triplicate samples were analyzed in each experiment. Data shown are the means of results obtained in at least *n* = 5–6 independent experiments. The error bars represent the standard deviations. Neutralization half maximal inhibitory concentration (IC_50_) was calculated by non-linear regression using the GraphPad Prism software. Statistical significance between LMHS and LMHS I629M was tested using a T test ( *, *P* < 0.05; **, *P* < 0.01).

**Fig 3:**
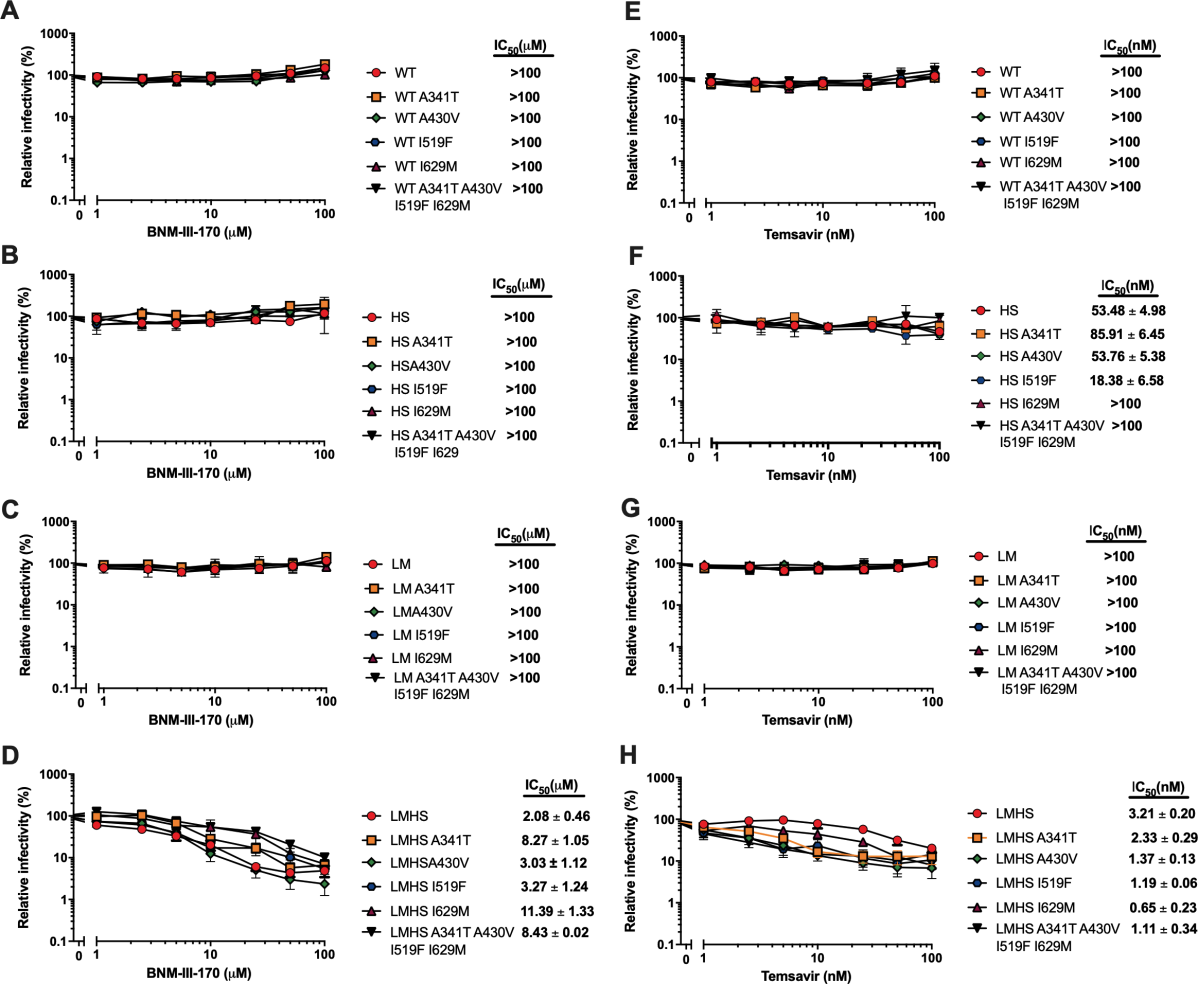
Effect of CM244 envelope variants on neutralization by a CD4mc or temsavir. Recombinant HIV-1 strains expressing luciferase and bearing wild-type or mutant CRF01_AE CM244 Envs were normalized by reverse transcriptase activity. Normalized amounts of viruses were incubated with different concentrations of BNM-III-170 (A–D) or temsavir (E–H) at 37°C for 1 h prior to infection of Cf2Th-CD4/CCR5 cells. Infectivity at each dilution of the CD4mc or temsavir is shown as the percentage of infection without the compound for each mutant. Triplicate samples were analyzed in each experiment. Data shown are the means of results obtained in at least *n* = 5–6 independent experiments. The error bars represent the standard deviations. Neutralization half maximal inhibitory concentration (IC_50_) was calculated by non-linear regression using the GraphPad Prism software.

To evaluate the impact of residues A341, A430, I519, and I629 on the susceptibility of Env to gp120 entry inhibitors, we mutated them alone or in combination with the LM mutations into the tier 2 CRF01_AE Env_CM244_. As expected from previous publications (19, 21), CRF01_AE WT or its H375S (HS) counterpart were resistant to both gp120 inhibitors, temsavir and the CD4mc BNM-III-170, but became sensitive in combination with the LM mutations (LMHS) (Fig. 2 and 3). We therefore mutated residues A341, A430, I519, and I629 within the H375S and LMHS backbones. While mutation of residues 341, 430, and 519 alone or in combination did not alter the susceptibility of HS or LMHS pseudoviral particles to these inhibitors (Fig. 3A through C and E through G), the I629M change did. Introduction of a methionine at position 629 of CM244 Env increased resistance to the CD4mc BNM-III-170 by approximately fivefold and showed a concomitant fivefold increase in the susceptibility to temsavir (Fig. 2A, B, 3D and H). The LMHS I629M mutant was the only variant to present opposite phenotypes against temsavir (increased susceptibility) and CD4mc (increased resistance) and was therefore selected for further analysis. However, we note that other mutants, such as LMHS A341T, presented some resistance to BNM-III-170 CD4mc.

Altogether, these results show that HR2 I629 has an effect on gp120 as illustrated by an approximately fivefold change in the susceptibility to temsavir and BNM-III-170. Of interest, the mutation had an opposite effect with the two inhibitors; it made the virus more susceptible to the conformational blocker and caused a concomitant increase in resistance to the BNM-III-170 CD4mc.

### I629M stabilizes the Env trimer

**Fig 4:**
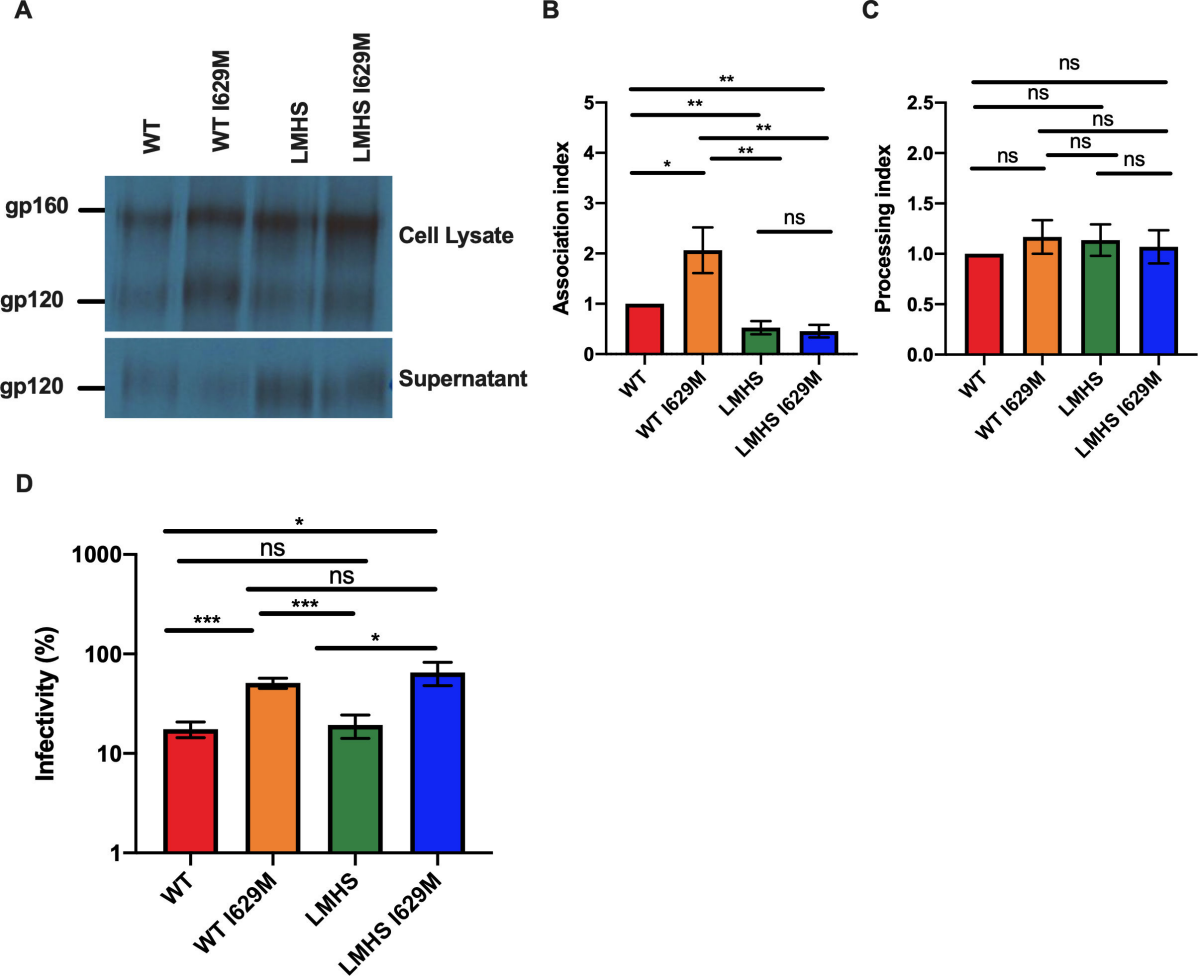
Effects of CM244 envelope variants on processing, subunit association, and sensitivity to cold inactivation. Cell lysates and supernatants (SN) of ^35^S-labeled cells transiently expressing the HIV-1 CM244 wild type, and the indicated mutant envelope glycoproteins were precipitated with plasma from PLWH. The precipitated proteins were loaded onto polyacrylamide gels (A) and analyzed by autoradiography and densitometry (B, C). Association index (B) and processing index (C) values of each envelope, calculated as described in the Materials and Methods. (D) Pseudoviruses bearing either wild type or mutant CM244 were incubated on ice for 72 h and infectivity was assessed using Cf2Th-CD4/CCR5 cells. Data represent the averages standard errors of the means (SEM) from four to five independent experiments. Statistical significance was tested using the t test (ns, not significant; *, *P* < 0.05; **, *P* < 0.01, ***, *P* < 0.001).

It has been described that Env conformation affects its susceptibility to small molecule inhibitors. Indeed, “closed” Envs tend to be more stable and susceptible to conformational blockers, while “open” Envs are more susceptible to molecules that prematurely trigger Env into downstream conformations (34, 35). To evaluate whether I629M stabilizes Env, we used a well-established assay that measures the ability of the mutant gp120 to remain associated with the Env trimer complex on the expressing cell, relative to that of the wild-type Env trimers (1, 36). Briefly, HEK293T cells were transfected with wild-type (wt) or mutant CRF01_AE CM244 Envs. Twenty-four hours post-transfection, cells were labeled with ^35^S-methionine/cysteine for 16 h as described in Materials and Methods. Cell lysates and supernatants were recovered and precipitated with a mixture of plasma from people living with HIV (PLWH). We found that the I629M mutant presented a significant increase (approximately twofold) in trimer association, as compared to its wild-type counterpart (Fig. 4A and B). A similar increase in viral infectivity was observed (Table 1), but whether these two phenotypes are associated remains to be determined. Of note, no differences in Env processing (Fig. 4C) or its capacity to mediate cell-to-cell fusion (Table 1) were observed. Finally, since Env stability governs the susceptibility of viral particles to inactivation by prolonged exposure to cold (0°C) (24, 34, 35, 37–40), we then asked whether the I629M affected cold inactivation. In this assay, prolonged incubation on ice decreases Env potential energy and results in functional inactivation. “Open” Envs are therefore more susceptible to cold inactivation, whereas more stable and “closed” Envs are resistant. Introduction of the I629M change into either CM244 or its LMHS counterpart similarly increased resistance to cold inactivation by more than threefold (Fig. 4D; Table 1). Altogether, these results suggest that I629M induces a change in Env, resulting in its stabilization, thus enhancing the susceptibility to temsavir and resistance to CD4mc.

**TABLE 1:** CRF01_AE CM244 envelope variant characterization

Envelope glycoprotein	Processing index^*a*^	Association index^*a*^	Relative infectivity^*a*^	Relative cell-cell fusion^*a*^	Cold t_1/2_ (hrs)
WT	1	1	1	1	16.99 ± 2.19
WT I629M	1.169 ± 0.17	2.065 ± 0.45	2.63 ± 0.62	0.8475 ± 0.09	55.14 ± 9.97
LMHS	1.137 ± 0.16	0.5252 ± 0.13	0.08397 ± 0.002	0.6537 ± 0.09	20.57 ± 5.62
LMHS I629M	1.07 ± 0.17	0.4574 ± 0.12	0.246 ± 0.038	1.039 ± 0.11	71.16 ± 29.76

^*a*^ Env processing index, subunit association index, cell–cell fusion and viral infectivity are shown for the WT and mutant variants of CM244 Envs and normalized to the values of WT CM244 Env.

### I629M stabilizes the interprotomer of gp120–gp41

**Fig 5:**
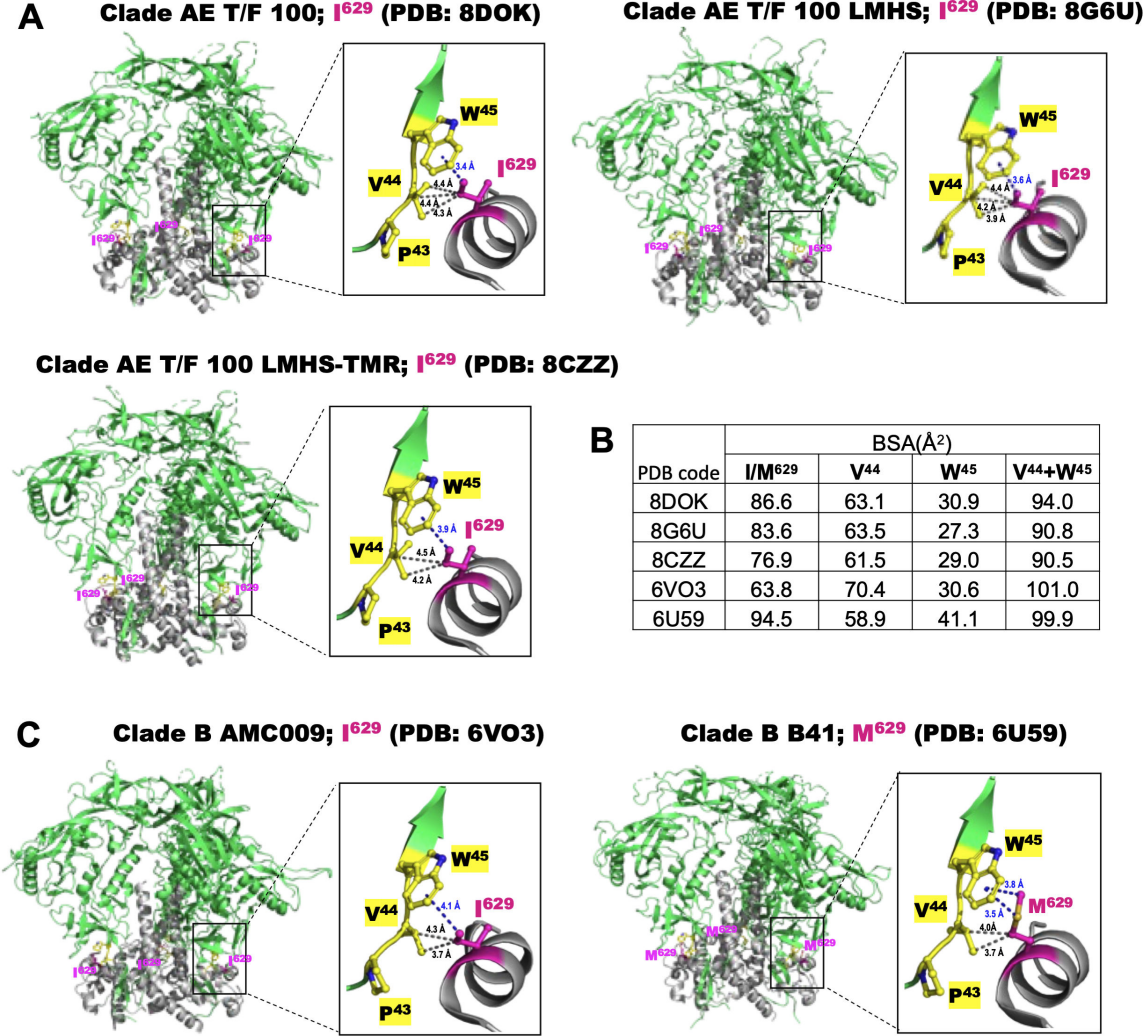
Interaction network formed by Ile/Met^629^ and gp120 residues within the HIV-1 Env trimer. (A) SOSIP trimers of clade AE T/F 100 wild-type (PDB: 8DOK), its LMHS mutant in the apo state (PDB: 8G6U), and temsavir-bound (PDB: 8CZZ) forms are shown alongside (C) SOSIP trimers of selected clade B strains, e.g. AMC009 (I629, PDB: 6VO3) and B41 (M629, PDB: 6U59). The trimers are represented as ribbons, with gp120 and gp41 protomers colored green and gray, respectively. Ile/Met^629^ and the interacting gp120 residues are depicted as sticks and are colored magenta and yellow, respectively. Close-up views highlight the details of the interprotomer interaction network formed by Ile/Met^629^, located within the gp41 subunit and the gp120 residues. The distances between Ile/Met^629^ and the benzene ring of Trp^45^, as well as the side-chain atoms of Val^44^, are indicated with blue and gray dashed lines, respectively. Only distances equal to or less than 4.5 Å are shown. (B) The residue-resolved buried-surface-area (BSA) of Ile/Met^629^ and Val^44^Trp^45^ in the five structures of (A) and (C), as determined by PISA.

To explore potential explanations for the effects observed in CRF01_AE Env with the I629M mutation on small-compound sensitivity and trimer stability, we examined the localization and interaction network involving I629 within the Env trimer. For this analysis, we utilized structures of CRF01_AE T/F 100 SOSIP trimers recently resolved in our laboratory. These included the wild-type apo form, LMHS apo form, and a LMHS-temsavir-bound form, all resolved with I629 (19) (Fig. 5A and B). Additionally, to test the possible consequences of the I629M mutation on overall trimer stability, we selected and analyzed the structures of SOSIP trimers (in the untriggered state) from the only available pair of same-clade strains (e.g., clade B) with Ile or Met at position 629 (41, 42) (Fig. 5B and C). As shown in Fig. 4, residue 629 participates in a highly conserved network of gp41–gp120 interprotomer contacts, involving V44 and W45 of gp120 inner domain seven-stranded β-sandwich. In both CRF01_AE T/F 100 wild-type and LMHS apo trimers, I629 forms hydrophobic interactions with the side-chain carbons of V44 and the benzene ring of W45. The number of hydrophobic contacts and the distance to the benzene ring of W45 do not vary significantly between the wild-type and LMHS trimers, indicating that LMHS mutations have minimal impact on the I629 interaction network. Buried surface area (BSA) analysis of I629 in CRF01_AE T/F 100 wild-type and LMHS apo trimers are 86.6 and 83.6 Å^2^, respectively. The BSA for W45 in two structures are 30.9 and 27.3 Å^2^, respectively. On the other hand, previous studies have shown that temsavir binds to HIV-Env by stabilizing its “closed” conformation (43). In agreement with this, we observed no significant changes to the interaction network involving I629, except for slightly larger distances between I629 and V44/W45. Additionally, the BSA of I629 in the temsavir-bound structure is reduced to 76.9 Å², and the combined BSA for W45 is 29.0 Å².

Interestingly, in clade B SOSIP trimers with an isoleucine at 629, e.g. AMC009 (Fig. 5C), there are slightly longer distances between the I629 side chain and the benzene ring of W45, and fewer contacts with V44, primarily due to a different side-chain conformation. The BSA of I629 in clade B SOSIP is significantly smaller, measuring just 63.8 Å², while the BSA for Trp45 is 30.6 Å² (Fig. 5B). In contrast, in clade B B41, where I629 is replaced by a methionine, the substitution compensates for these weaker contacts. The extended side chain of M629 establishes more extensive interactions with W45, including hydrophobic interactions mediated by the terminal carbon and sulfur atom of M629. The BSA values for M629, V44, and W45 in this structure are much larger than those observed for AMC009, CRF01_AE T/F 100 wild-type, and LMHS apo trimers, with a BSA for M629 of 94.5 Å² and a BSA for W45 of 41.1 Å². These findings suggest that, in general, a methionine at position 629 stabilizes the interprotomer gp120–gp41 contacts in HIV-1 Env more effectively than an isoleucine. The P43–V44–W45 stretch is highly conserved among HIV-1 strains, and the interaction dynamics in this region likely favors longer, bulkier, or more hydrophobic residues at position 629. Such residues may enhance interprotomer stability, reducing susceptibility to CD4-induced opening.

## DISCUSSION

The HIV envelope undergoes a series of intricate conformational shifts, moving from a “closed” to an “open” state during viral entry (4). The most widely circulating HIV strains preferentially remain in a “closed” conformation; however, the CRF01_AE strain displays a unique structural dynamic. The presence of the bulkier amino acid histidine at position 375 within the Phe43 cavity predisposes Env to sample a more “open” conformation (20, 44) rendering Env more susceptible to non-neutralizing CD4-induced (CD4i) antibodies (1, 20, 45, 46). The filling of the Phe43 cavity with a histidine also renders CRF01_AE strains resistant to CD4mc inhibitors and to conformational blocker inhibitors, such as temsavir (19–21, 47, 48).

Further investigation unveiled that histidine alone is not the sole architect of this resistance. Six additional co-evolving residues within the gp120 layers have been identified that work synergistically to modulate temsavir resistance (19). Comparative sequence analysis of M subtypes from the NIH Los Alamos HIV database with CRF01_AE highlighted a distinct set of conserved residues unique to CRF01_AE. These residues are distributed across key regions: the gp120 domain, the interface of gp120 and gp41, and the HR2 region of gp41. Whether these residues co-evolved with position 375 to avoid potential detrimental Env recognition by CD4i Abs remains to be determined.

Functional studies involving mutations of these residues in various CM244 variants, both individually and in combination, underscored the critical influence of the I629M mutation in modulating resistance to CD4mc and cold inactivation and an increase in susceptibility to the conformational blocker temsavir.

Based on our structural interpretations, we postulate that the observed effects depend on the capacity of I629M to enhance the association between the gp120 and gp41 subunits. The stronger hydrophobic interactions mediated by M629 compared with I629 may contribute to maintaining a more “closed” Env conformation. This hypothesis aligns with our functional data showing an altered sensitivity to two classes of small-molecule Env inhibitors. CD4mc preferentially bind to “open” Env conformations and stabilize the CD4-induced conformation (11). In contrast, temsavir stabilizes the “closed” conformation of Env, preventing transitions to the CD4-bound state and thereby inhibiting viral entry (14, 19). These findings collectively suggest that the I629M mutation may differentially affect the binding and efficacy of these compounds by altering the conformational dynamics of Env. Our study also indicates that residues located far from their binding site modulate the susceptibility of Env to small molecule inhibitors. Since temsavir is in the clinic, our study raises the importance of monitoring for potential resistance mutations arising beyond the gp120 binding site, including in the gp41.

In summary, we demonstrate that a naturally occurring I629M substitution in HIV-1 gp41 leads to a more stable Env conformation, potentially enhancing the overall viral fitness by preserving the integrity of the envelope trimer.

## Data Availability

Data and reagents are available upon request.
